# Consistency of Targeted Metatranscriptomics and Morphological Characterization of Phytoplankton Communities

**DOI:** 10.3389/fmicb.2020.00096

**Published:** 2020-02-06

**Authors:** Kristiina Vuorio, Anita Mäki, Pauliina Salmi, Sanni L. Aalto, Marja Tiirola

**Affiliations:** ^1^Freshwater Centre, Finnish Environment Institute (SYKE), Helsinki, Finland; ^2^Department of Biological and Environmental Sciences, Nanoscience Center, University of Jyväskylä, Jyväskylä, Finland

**Keywords:** phytoplankton, high-throughput sequencing, microscopy, ribosomal RNA, freshwater

## Abstract

The composition of phytoplankton community is the basis for environmental monitoring and assessment of the ecological status of aquatic ecosystems. Community composition studies of phytoplankton have been based on time-consuming and expertise-demanding light microscopy analyses. Molecular methods have the potential to replace microscopy, but the high copy number variation of ribosomal genes and the lack of universal primers for simultaneous amplification of prokaryotic and eukaryotic genes complicate data interpretation. In this study, we used our previously developed directional primer-independent high-throughput sequencing (HTS) approach to analyze 16S and 18S rRNA community structures. Comparison of 83 boreal lake samples showed that the relative abundances of eukaryotic phytoplankton at class level and prokaryotic cyanobacteria at order level were consistent between HTS and microscopy results. At the genus level, the results had low correspondence, mainly due to lack of sequences in the reference library. HTS was superior to identify genera that are extensively represented in the reference databases but lack specific morphological characteristics. Targeted metatranscriptomics proved to be a feasible method to complement the microscopy analysis. The metatranscriptomics can also be applied without linking the sequences to taxonomy. However, direct indexing of the sequences to their environmental indicator values needs collections of more comprehensive sample sets, as long as the coverage of molecular barcodes of eukaryotic species remains insufficient.

## Introduction

Aquatic microbes, including prokaryotic phototrophic and mixotrophic bacteria (i.e., cyanobacteria or Cyanophyceae), and eukaryotic phytoplankton, are integral components of aquatic food webs. Using satellite observations, the net primary production of phytoplankton (45–50 Gt C year^−1^) has been estimated to be close to that of the land plants (45–68 Gt C year^−1^; Longhurst et al., 1995). Phytoplankton communities are composed of relatively short-lived organisms that are dependent on the surrounding water. Both the composition and diversity of the phytoplankton community respond rapidly to changes in water quality and to physical and meteorological forcing (Cole, 1982; Reynolds, 2006). Therefore, the composition of the phytoplankton is an excellent indicator of environmental disturbances (Sommer et al., 1993; Reynolds, 2006), and can be used to monitor and evaluate the ecological status of aquatic ecosystems (e.g., Padisák et al., 2006; Carvalho et al., 2013).

The previous information on the composition and diversity of the phytoplankton community has been obtained with the optical microscopy (Utermöhl, 1958; Willén, 1976; EN 15204, 2006). Conventional light microscopy analyses are mainly based on cell morphology, assuming that phytoplankton taxa have structural features that allow species identification (e.g., Reynolds, 2006). Light microscopy has a limited ability to discriminate between species, which leads to underestimation of diversity, and it may also falsely interpret different morphotypes of the same species into distinct species. These disadvantages are not only related to morphological plasticity (cryptic species) and size (picoplankton), but also to changes in taxonomic detection limits and changes in nomenclature, due to improved research methods and more detailed taxonomic knowledge. In addition, conventional 2–50 ml sample volumes and microscopy techniques, where only a fraction of the volume is studied, can severely underestimate species richness in environmental samples (Cermeño et al., 2014; Rodriguez-Ramos et al., 2014).

There is an urgent need to develop and evaluate more accurate, and cost-efficient methods to replace traditional optical microscopy-based phytoplankton analyses to discover the true aquatic microbial diversity. Molecular-based methods could provide rapid, inexpensive, and more accurate identification of aquatic organisms (e.g., McManus and Katz, 2009). The development of high-throughput sequencing (HTS) platforms and applications enables the economical sequencing of millions of genomic fragments extracted from environmental samples (e.g., Hajibabaei et al., 2011; Porter and Hajibabaei, 2018). Indeed, the sequencing of small subunit ribosomal RNA (SSU rRNA) genes is becoming a widespread tool for identifying and characterizing organisms (e.g., Jost et al., 2010; Krienitz and Bock, 2012) and environmental monitoring (e.g., Shokralla et al., 2012; Valentini et al., 2016; Hering et al., 2018). In phytoplankton studies, the introduction of molecular tools has changed the classification of many groups, both in their phylogenetic position (i.e., change in a species or generic designation for a given taxon) and in taxonomic rearrangements and definition of new taxa (e.g., Komárek, 2010). Recent introduction of barcoding, i.e., indexing of samples and multiplexing, combined with HTS provides new procedures for community ecology, and thus new perspectives in the microbial community studies (Cristescu, 2014).

In HTS, DNA fragments of taxonomic marker genes can be obtained by amplifying with gene-specific PCR primers (Siegwald et al., 2017), or by using untargeted shotgun metagenomics to recover complete genome sequences (Quince et al., 2017). In traditional SSU rRNA amplicon sequencing using gene-specific primers, only 10% of the environmental microbial sequences can be detected, and the abundance of certain groups (e.g., members of the Candidate Phyla Radiation and archaea) can be overestimated (Eloe-Fadrosh et al., 2016). In addition, archaea and eukaryotes lack good universal rRNA primers due to the lack of reference libraries (Klindworth et al., 2013), which prevents simultaneous amplification of all domains of life. Since shotgun sequencing is not a resource-efficient way to estimate diversity in large microbiome studies, 0.5 million reads being the minimum number of required sequences even for shallow metagenomics (Hillmann et al., 2018), other cost-efficient options are needed.

Although most DNA is expected to be found in living cells, long-lasting DNA in damaged cells or soluble extracellular or relic DNA can, e.g., in soil form 40% of total DNA (Carini et al., 2017). On the contrary, RNA is only present in living organisms (and viruses) with active protein synthesis. Ribosomal RNA is an integral structural component of ribosomes, accounting for the majority (82–90%) of cellular RNA (Neidhardt and Magasanik, 1960). RNA is rapidly degraded due to ubiquitous presence of intracellular and extracellular RNA-degrading ribonuclease enzymes (RNases) (Houseley and Tollervey, 2009). Structural features, such as the single-stranded form and an additional hydroxyl group in the ribose sugar component, also make RNA molecule more labile than the DNA molecule (Lodish et al., 2000). Although RNA-based library preparation for HTS is challenging, RNA-based results from a community composition of eukaryotic species are more realistic than DNA-based results due to the enormous amount of rRNA gene copy number variations between phytoplankton species. When the relative abundances of phytoplankton strains were studied in our earlier study, the results of DNA-based amplicon sequencing did not correlate with the phytoplankton biomasses, whereas the results of rRNA-based analyses were more consistent with biomass indicators (Mäki et al., 2017).

Primer-independent sequencing of SSU rRNA is complicated, since it is presumed that prokaryotic RNAs and mature eukaryotic rRNAs lack poly(A) tailing, which would facilitate direct reverse transcription (Slomovic et al., 2006). Botero et al. (2005) developed a method to add poly(A) tails to environmental RNA, and Karst et al. (2018) even doubled the known diversity of micro-organisms represented in the curated SILVA SSU database when studying microbiome samples of seven environments using the approach combined with synthetic long-read Illumina sequencing. In addition to poly(A) tail use, the RNA-based HTS library construction may also be based on ligation of sequencing adapters to enable library amplification (Linnarsson, 2010). In a novel library construction method, ligation of the RNA oligo (M13) to the 5′-end of the rRNAs enabled simultaneous primer-independent HTS of rRNAs from prokaryotic and eukaryotic species (Mäki and Tiirola, 2018).

In this study, we compared the results of the phytoplankton community composition obtained by the novel HTS method (Mäki and Tiirola, 2018) with conventional light microscopy, and evaluated the coverage of the HTS method. The major advantage of simultaneous sequencing of 16S and 18S rRNA is that it allows the detection of all phytoplankton taxa, i.e., prokaryotic cyanobacteria and eukaryotic phytoplankton without bias due to copy number variation and unequal primer match. The use of the 83 lakes dataset indicated that, in the absence of sequences in the reference databases, the number of shared taxa obtained by both methods was small. In addition, the relative abundances of sequences corresponded poorly to the wet weight biomass of taxa obtained by microscopy. However, the proportions of eukaryotic phytoplankton class-level results and cyanobacterial order-level results were similar, indicating the potential of the HTS method used.

## Materials and Methods

**Figure 1:**
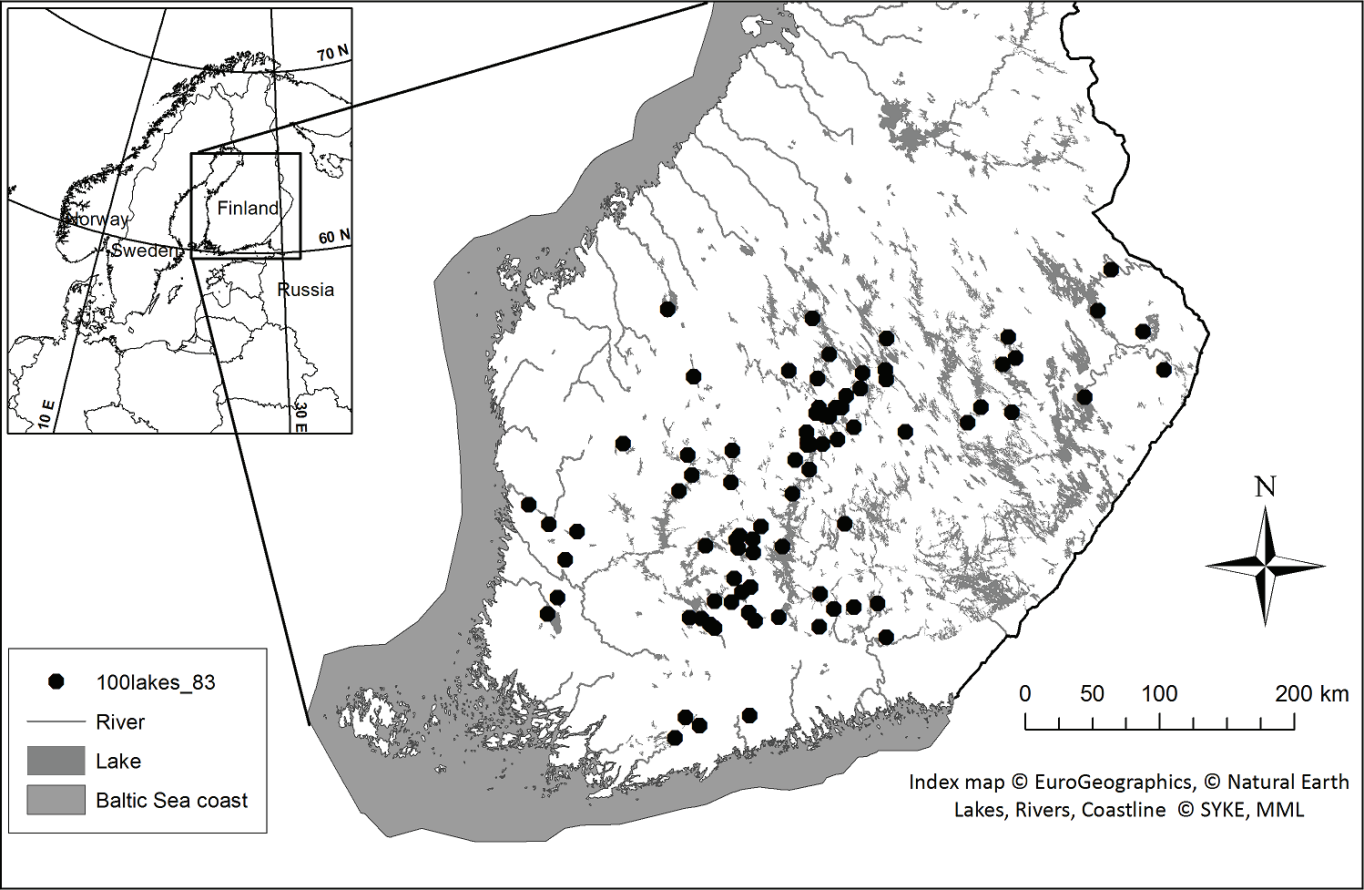
Sampling sites in Southern and Central Finland during late June–August in 2014 and 2015.

For phytoplankton microscopy and molecular analyses, surface water samples were collected from 83 lakes during June–August 2014 and 2015 in southern and central Finland (Figure 1). Integrated samples were collected with a 0.5 or 0.3 m long water sampler (Limnos Ltd., Finland) from the surface down to a depth of 2 m (0–2 m). In cases where the thermocline was above the 2-m depth (i.e., the difference in water temperature was more than 1°C within 1 m), the samples represent depth from the surface down to the beginning of the thermocline, e.g., 1 m (0–1 m). Water was collected into a 40 L plastic bucket, mixed gently and pre-sieved through a 250 μm mesh to remove larger zooplankton.

Water samples for molecular analysis were taken to the laboratory in 5-L plastic canister, which were kept cool (4–8°C) during transport. From each sample, 50–250 ml of lake water was filtered onto a water filter (MoBio, US, diameter 47 mm, nominal pore size 0.22 μm) with a maximum vacuum of 75 mm Hg (10 kPa). Filters were kept at −80°C until analyzed. The phytoplankton samples for light microscopy analyses were taken into 100 ml clear polyethylene terephthalate bottles and preserved immediately with acid Lugol solution (0.5 ml Lugol per 100 ml), and then stored in refrigerator.

Picophytoplankton samples for epifluorescence microscopy were prepared by filtering water from the 5 L canister first through sterile 5 μm pore size syringe filters and then filtering 5 ml of the syringe-filtered water onto black polycarbonate filters (pore size 0.22 μm, Merck Millipore, Germany) using the vacuum filtration system. The black filters were mounted between object and cover glasses with glycerol and stored at −20°C. Picophytoplankton samples were prepared from 69 of the 83 lakes.

### Light Microscopy

Phytoplankton microscopy analyses were performed according to Utermöhl technique (Utermöhl, 1958; EN 15204, 2006) using an inverted microscope (Leitz Labovert FS) with phase contrast illumination. Subsamples of 3, 10, 25, or 50 ml were settled in Utermöhl settling chambers for at least 24 h prior to analysis. Three different total magnifications, 125, 250, and 1,000X, were used to cover all phytoplankton taxa, regardless of their size. A total of 14–125 and 12–100 fields of view were randomly selected from two transects, perpendicular to each other, with the two higher magnifications of 1,000 and 250X, respectively. The proportion of the total area of the settling chamber, counted with the two highest magnifications, was 0.1–1.2% and 1.8–14.8% of the bottom area of the settling chamber. The entire bottom area of the settling chamber was counted with the lowest magnification 125X. At least 400 units were counted with each magnification wherever possible. Phytoplankton were identified at species level by morphology where possible. Cell numbers were converted to wet weight biomass according to an appropriate geometric formula (Hillebrand et al., 1999; EN 16695, 2015) and by assuming cells to be isopycnal with water.

### Epifluorescence Microscopy

Counting of picophytoplankton samples was based on autofluorescence of the cells (MacIsaac and Stockner, 1993). An Axio Vert.A1 epifluorescence microscope (Carl Zeiss, Germany) equipped with a blue (470 nm) and a green (530 nm) LED excitation light source was used for the counting. Phycoerythrin-rich picocyanobacteria emitted bright orange and phycocyanin-rich picocyanobacteria emitted bright red autofluorescence with filter set 14 (EX: BP 510-560, beamsplitter: 580, EM: LP 590, Carl Zeiss, Germany). Chloroplasts of eukaryotic picophytoplankton emitted distinguishable red autofluorescence with filter set 09 (EX: BP 450-490, beamsplitter: 510, EM: LP 515, Carl Zeiss, Germany). Picophytoplankton were counted with 1,000X magnification from randomly selected fields of view until a confidence interval of the mean biomass was 30% (Salmi et al., 2014), but at least 10 fields of view were counted. The wet weight biomass of the picophytoplankton was calculated by approximating the cell biovolume (sphere, rotational ellipsoid, or cylinder). The main dimensions were measured with an eyepiece grid of 1 μm scale. Cell volumes were converted to wet weight biomass with the assumption that the cells were isopycnal with water.

### RNA Extraction, Library Preparation, and Sequencing

The previously developed primer-independent HTS method (Mäki and Tiirola, 2018) was used. The time between sampling and sample processing took 3–4 years, due to the time needed to develop the new sequencing procedure. Prior to RNA extraction, each frozen filter was aseptically cut in half, and the halves were placed in separate 2 ml tubes containing 0.1 mm glass beads (Omni, International, US) and filled with 400 μl of TRI Reagent (Zymo Research, US). To lyse the cells, bead beating was done with Power Lyse 24 homogenizer at 3400 RPM for 40 s. After centrifugation for 1 min at 12000 × *g*, the supernatants from both tubes were pooled resulting in one tube per sample. RNA extraction was continued using Direct-Zol RNA MicroPrep isolation kit (Zymo Research, USA) according to the manufacturer’s instructions, including DNA digestion. The RNA yield ranged from 0.07 to 7.73 ng ml^−1^.

The library preparation was done as described by Mäki and Tiirola (2018). Briefly, RNA extractions were run in a precast 1% agarose E-Gel EX gel (Invitrogen, US) and 16S/18S rRNA fragments were cut from the gel using a flamed scalpel and then purified using the Zymoclean Gel RNA Recovery Kit (Zymo Research, US). M13-RNA oligo (5′-UGUAAAACGACGGCCAGU-3′) was ligated to the 5′-end of the RNA fragments using T4 RNA ligase (Promega, US). The ligation product was purified using Agencourt RNAClean XP (Beckman Coulter, US) system. Synthesis of cDNA was performed using a random primer with a P1 sequencing adapter overhang (5′-CCTCTCTATGGGCAGTCGGTGATNNNNNN-3′). After RNAClean purification of the cDNA, amplification was done using a barcoded Ion Torrent sequencing adapter as a forward primer (IonA sequence with a barcode and M13-sequence in the 3′-end) and P1 as a reverse primer. The dual size-selection procedure of the ProNex Size-Selective Purification System (Promega, US) and synchronous purification of amplicons was run in one step, targeting the selection between 300 and 550 base pairs. RNA and amplification products were analyzed using the Agilent 2200 TapeStation system (Agilent, US) with High Sensitivity RNA ScreenTape and the High Sensitivity D1000 ScreenTape Assay, respectively. HTS was performed with the Ion Torrent Personal Genome Machine (Thermo Fisher Scientific, US) using the Ion PGM Hi-Q View OT2 400 kit, the Ion PGM Hi-Q View Sequencing kit (quality control included), and the Ion 316v2 chip. The sequencing data were initially trimmed with Torrent Suite 5.10.0 software with IonA and P1 adapter sequence removal and polyclonality filtering. The default 3′-end quality trimming of the Torrent Suite application was turned off (command: “--trim-qual-cutoff 100”).

#### Sequence Processing and Bioinformatics

Fastq-files were deposited in the National Center for Biotechnology Information (NCBI) database (SRA accession PRJNA577554). The data were imported into CLC Genomics Workbench 11.0.1 software^1^ and trimmed using a modified-Mott trimming algorithm with an error probability limit of 0.05. The M13-adapter and 10 nucleotides were removed from the 5′-end. Removal of the nucleotides was done to avoid penalizing the alignment scores due to unaligned ends, when the input reads were longer than the database reference sequences. The minimum number of nucleotides was imposed to 150. The SILVA v132 16S/18S reference rRNA gene database was used for picking Operational Taxonomic Units (OTUs; 97% similarity between OTUs) and for taxonomic assignment. The chimera crossover cost was set to 3 and k-mer size was set to 6, the minimum occurrence was set to 1. Creation of new OTUs was allowed and the *de novo* OTU taxonomy similarity percentage was set to 90.

Because of the method used, the resulting sequences did not necessarily start from a certain point of the 5′-end of rRNA genes. Since the positional homology of the fragments was lost, several OTUs represented the same identity. Therefore, the aligned cyanobacterial and eukaryotic phytoplankton sequences were grouped according to their taxonomy to reduce the number of sequences referring to the same identity. The identities, referred as taxa, represent the level of taxonomy each sequence was identified (i.e., phylum, class, order, genus, or species). Comparisons of the HTS results with the light microscopy results are based on the taxonomy used in Algaebase (Guiry and Guiry, 2018).

### Diversity Indices and Statistical Analyses

The differences in the species richness (S), Shannon diversity (H′), and Evenness (E) indices were compared between light microscopy and HTS using a Related-samples Wilcoxon signed rank test. Species richness was expressed as the total number of taxa per sample. The Shannon diversity index takes into account the relative abundances of species. It was calculated as follows: H′ = Σp_i_ * *ln* p_i_, where p_i_ is the relative abundance of taxon i. Evenness E = H′/ln(S).

Statistical analyses were performed using R version 3.4.3 (R Core Team, 2018) and IBM SPSS Statistics (version 24). The results of the HTS analyses of the 83 samples were compared with the microscopy analysis results of the same samples, and Mantel tests were performed to examine phylum and class-level community matrix correlations obtained with HTS and light microscopy. Related-samples Wilcoxon signed rank test was performed to test for differences between proportions of cyanobacterial sequences obtained by HTS and wet weight biomass obtained by light microscopy. An arcsine transformation was performed to meet the test requirements.

## Results

### Light Microscopy

Phytoplankton larger than picoplankton (>2 μm in size), representing nine different phyla (cyanobacteria, Cryptophyta, Dinophyta, Ochrophyta, Diatomophyta, Haptophyta, Euglenophyta, Chlorophyta, and Charophyta), were included in the microscopy analyses. A total of 626–2,608 different phytoplankton counting units (i.e., solitary cells, filaments, coenobia, or colonies) were counted per sample under the light microscope. Taking into account the number of cells in each counting unit (filament, coenobia, and colony), the total number of cells in the samples ranged from 2,375 to 71,585. A total of 484 phytoplankton taxa were identified by light microscopy based on morphological features. Of the taxa, 98 were prokaryotic cyanobacteria, and the remaining 386 were eukaryotic phytoplankton. A total of 369 taxa could be identified at species level, representing nine phyla, 22 classes, and 196 genera.

### Epifluorescence Microscopy

Solitary cells (<2 μm in size) and microcolonies were counted as picophytoplankton. Most picophytoplankton (mean 83% of the wet weight biomass, min 32%, max 100%) were picocyanobacteria. Phycocyanin-rich picocyanobacteria were the most common types, with an average proportion of 93% (min 10%, max 100%) of the wet weight biomass of picocyanobacteria.

**Figure 2:**
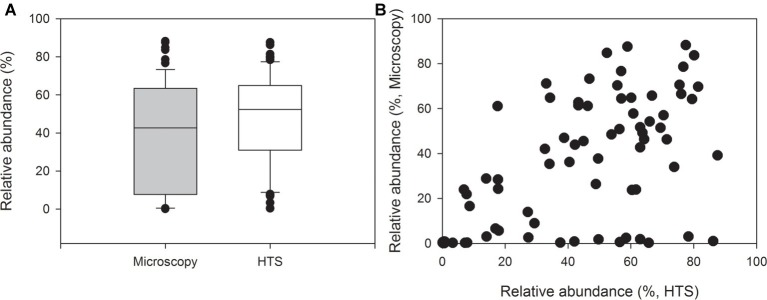
Relative abundances of picocyanobacteria of all cyanobacteria in 69 lake samples. The graph shows **(A)** median, standard deviation, 25 and 75% percentiles as well as outlier values of relative abundances and **(B)** relative abundances obtained by high-throughput sequencing (HTS, *x*-axis) and biomass analysis obtained by epifluorescence microscopy (wet weight, *y*-axis).

Picocyanobacteria comprised on average 39% of the total cyanobacterial biomass (min 0.01%, max 88%, Figure 2A). This was rather consistent with the proportion of picocyanobacterial sequences of the total number of cyanobacterial sequences (average 48%, min 0.4%, max 88%, Figures 2A,B). However, the difference between the proportions of picocyanobacterial sequences obtained by HTS and their proportions of the wet weight biomass obtained by epifluorescence microscopy was significant (Related-samples Wilcoxon signed rank test, *p* = 0.024).

### High-Throughput Sequencing

The HTS results included taxa from all the three domains of life, i.e., Archaea, Eukarya, and Bacteria. After quality and chimeric reads trimming, the dataset contained 2,320,565 reads and 199,022 OTUs (OTU clustering at 97% similarity). The number of OTUs ranged from 1,333 to 11,141 per sample, and the number of reads (assigned to OTUs) ranged from 9,661 to 57,222 per sample. Of the reads, a total of 60,195 (30.2%) could be aligned to SSU rRNAs based on the SILVA database. After deletion of chloroplast and mitochondrial read sequences and unaligned sequences (which could not even be assigned at domain level), the remaining 53,059 OTUs could be divided into Archaea (10 OTUs), Bacteria (46,767 OTUs, incl. cyanobacteria), and Eukaryota (6,282 OTUs, incl. plants, metazoans, protists, and fungi). Although HTS analysis was able to detect all organism groups, we focused on phytoplankton, including prokaryotic cyanobacteria (Cyanophyceae) and eukaryotic phytoplankton.

Of the phytoplankton sequences obtained by HTS, 16,876 OTUs represented cyanobacteria, of which 9,014 were picoplankton. Only 2,515 eukaryotic phytoplankton OTUs were obtained, and only four of them represented picoplankton. Several reads represented cultured strains that are found several times in the SILVA database with different accession numbers, artificially increasing the number of OTUs. Due to degradation of the 5′-end of the rRNAs and the method used, the starting and ending points of the sequences varied, and sequence alignment resulted in several OTUs representing the same taxon. To minimize the number of these artificial OTUs, OTUs were grouped based on their taxonomic identity, hereafter referred as taxa representing species, genera, or higher taxonomic levels. After this grouping, HTS was able to separate 566 different taxa, representing nine phyla and 34 classes. Of these taxa, 164 represented different cyanobacterial taxa, while 402 represented different eukaryotic phytoplankton, which belonged to the same nine phyla as taxa identified by microscopy analysis.

### Comparison of Microscopy and High-Throughput Sequencing Results

**Figure 3:**
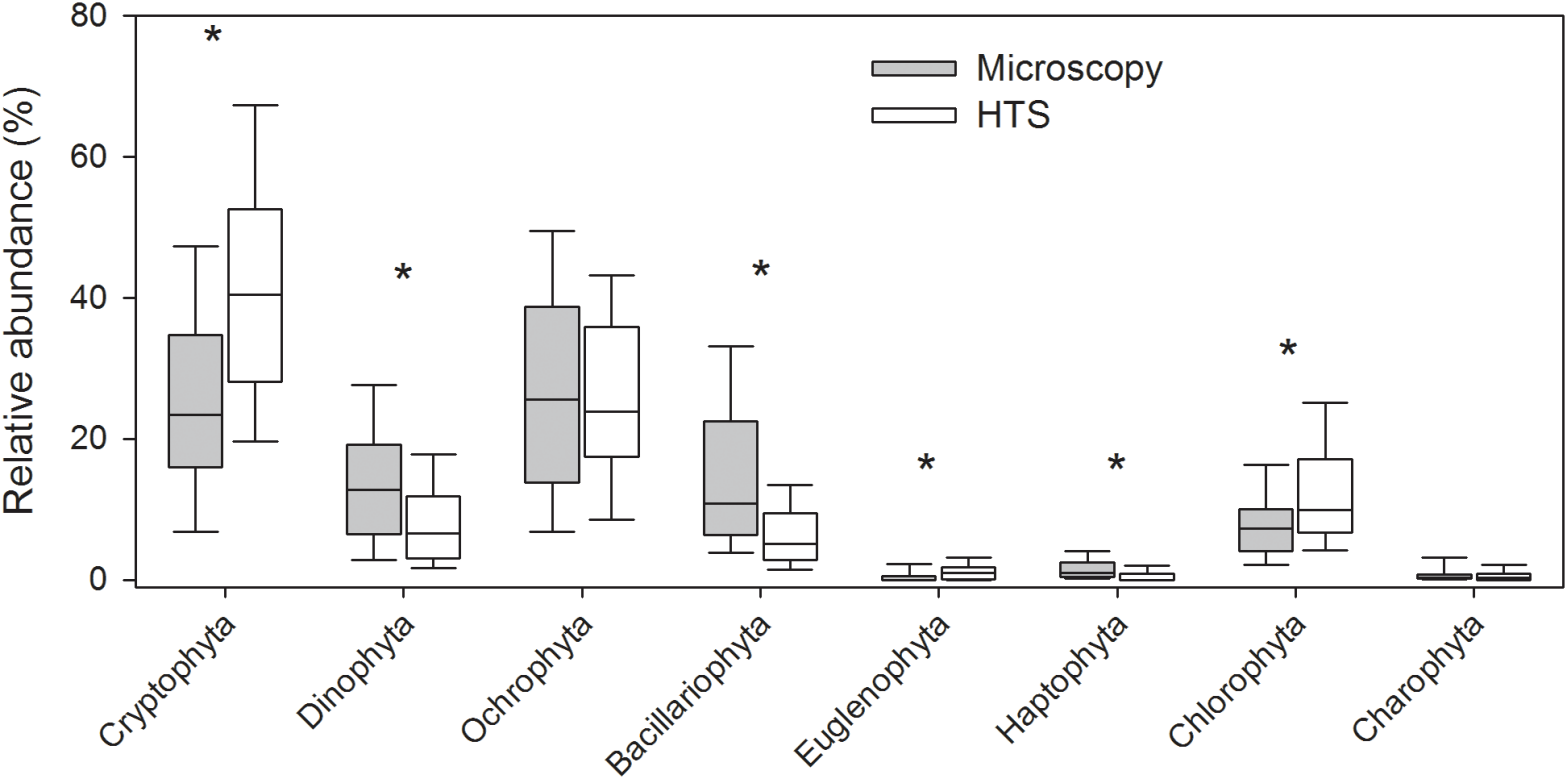
Relative abundances of eukaryotic phytoplankton at phylum level obtained by high-throughput sequencing (HTS) and light microscopy. The graph shows median, standard deviation, and 25 and 75% percentiles of relative abundance values over the whole set of 83 lake samples. Significant differences between the light microscopy and HTS results are indicated with an asterisk (^*^) (Related-samples Wilcoxon signed rank test).

**Figure 4:**
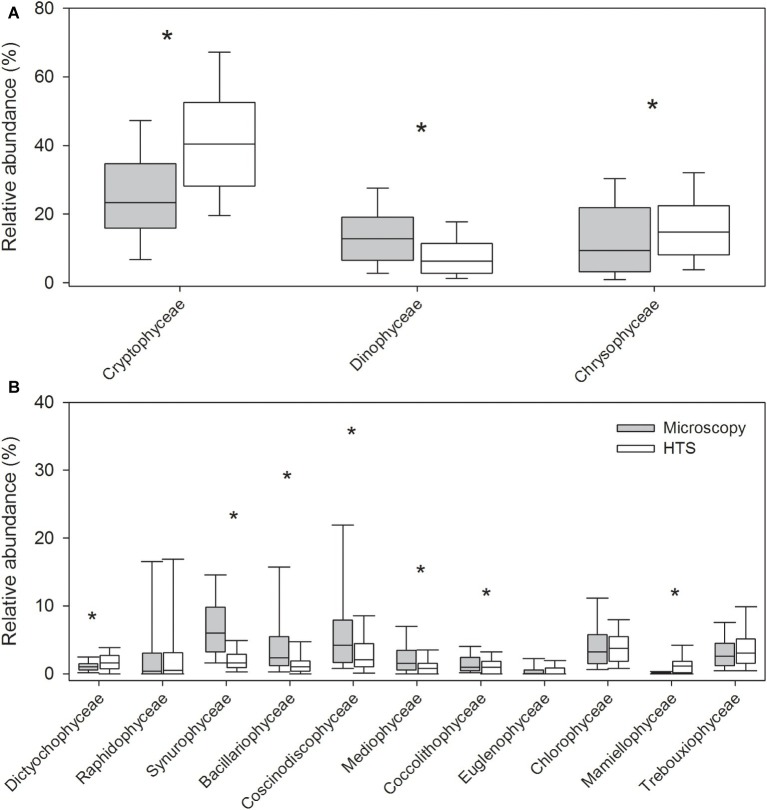
Relative abundances of the most abundant eukaryotic phytoplankton at class level obtained by high-throughput sequencing (HTS) and light microscopy. The graph shows median, standard deviation, and 25 and 75% percentiles over the whole set of 83 lake samples. The classes belong to the following phyla in parenthesis: **(A)** Cryptophyceae (Cryptophyta), Dinophyceae (Dinophyta), and Chrysophyceae (Ochrophyta), and **(B)** Dictyochophyceae, Raphidophyceae, and Synurophyceae (Ochrophyta), Bacillariophyceae, Coscinodiscophyceae, and Mediophyceae (Bacillariophyta), Coccolithophyceae (Haptophyta), Euglenophyceae (Euglenophyta), as well as Chlorophyceae, Mamiellophyceae, and Trebouxiophyceae (Chlorophyta). Significant differences between the light microscopy and HTS results are indicated with an asterisk (^*^) (Related-samples Wilcoxon signed rank test).

The mean proportion of cyanobacteria in HTS reads was significantly higher (93%) than the one determined by microscopy (23%). At phylum level, the same eight eukaryotic phyla were identified with both the methods (Figure 3, Supplementary Figure S1). The community matrices obtained by the two methods were similar (Mantel test between Bray-Curtis dissimilarity matrices; *r* = 0.25, *p* = 0.001). However, the differences between the methods were significant for most phyla, except for Ochrophyta, and Charophyta (Related-samples Wilcoxon signed rank test, *p* < 0.001; Figure 3). HTS was able to detect all phytoplankton classes identified by light microscopy. At class level, HTS was also able to detect non-planktonic classes that are not usually included in light microscopy analysis but species belonging to these classes may occasionally occur in plankton. These classes represented filamentous periphytic/epiphytic taxa (Ulvophyceae, Coleochaetophyceae), which are meroplanktonic and therefore are not routinely included in light microscopy phytoplankton analysis. Taxa lacking morphological characteristics (which are required even for class-level identification by light microscopy; e.g., Mesostigmatophyceae, Mamiellophyceae) were identified only by HTS. Some of the classes identified by HTS represented marine taxa (e.g., Pavlovophyceae, Phaeothamniaceae, and Pinguiophyceae) or terrestrial taxa (Chlorokybophyceae). It is also possible that by light microscopy, some taxa may not have been recognized as phytoplankton (e.g., Synchromatophyceae), or may have been confounded with another taxon, and therefore have been identified incorrectly (e.g., Chloromonadophyceaean genus *Tetraselmis* and Pyramimonadophyceaen genus *Pyramimonas*). However, both methods resulted in similar eukaryotic class-level community matrices (Mantel test, *r* = 0.25, *p* = 0.001), but the differences between the methods were significant for most classes, except Raphidophyceae, Euglenophyceae, Chlorophyceae, and Trebouxiophyceae (Related-samples Wilcoxon signed rank test, *p* < 0.021; Figure 4).

**Figure 5:**
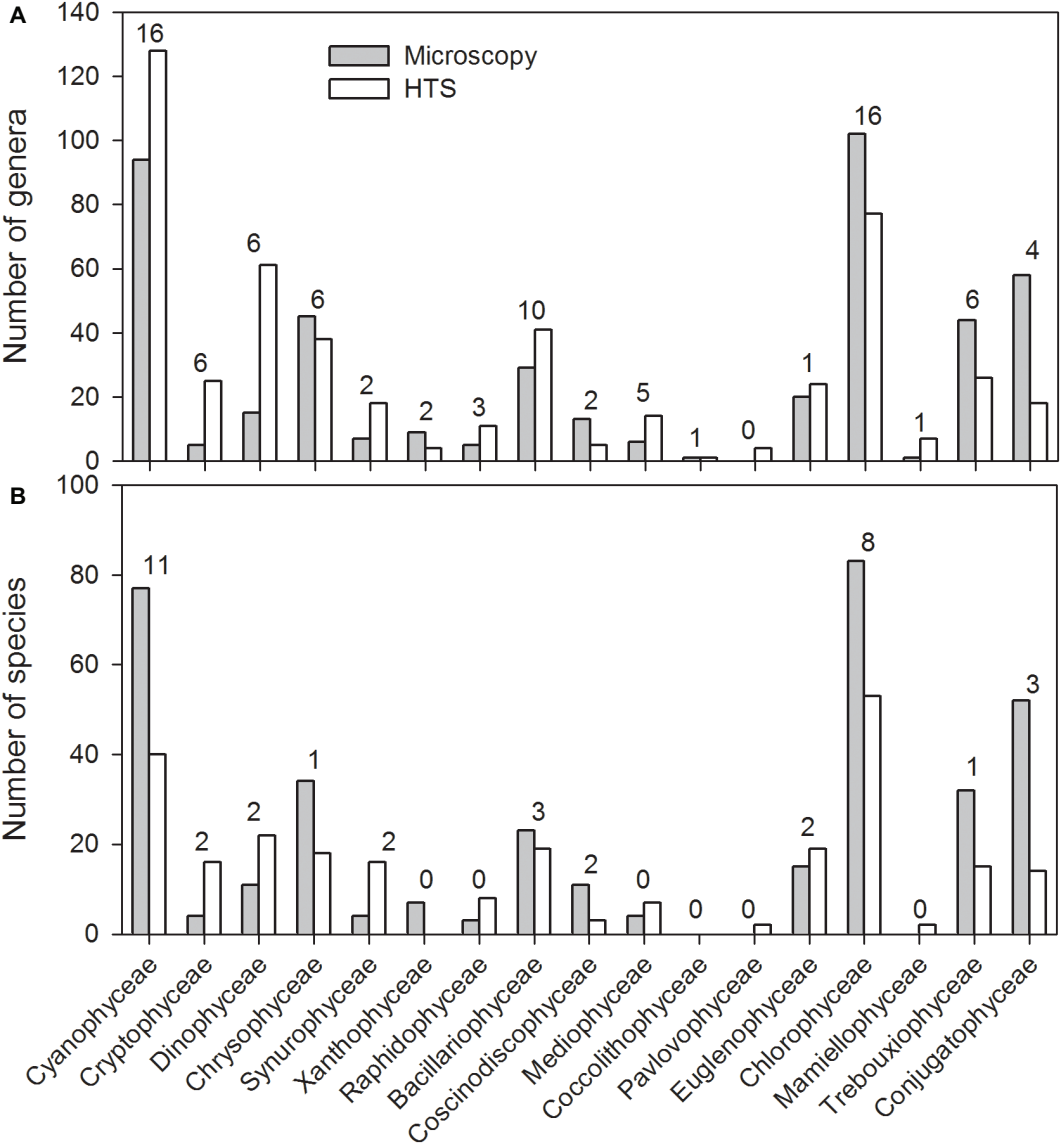
Total number of genera **(A)** and species **(B)** within the most abundant phytoplankton classes observed in the 83 lake water samples based on high-throughput sequencing (HTS) and light microscopy analysis. The phyla in which the classes belong to are presented in the caption of Figure 4. The numbers above the columns indicate the number of species obtained by both light microscopy and HTS.

**Figure 6:**
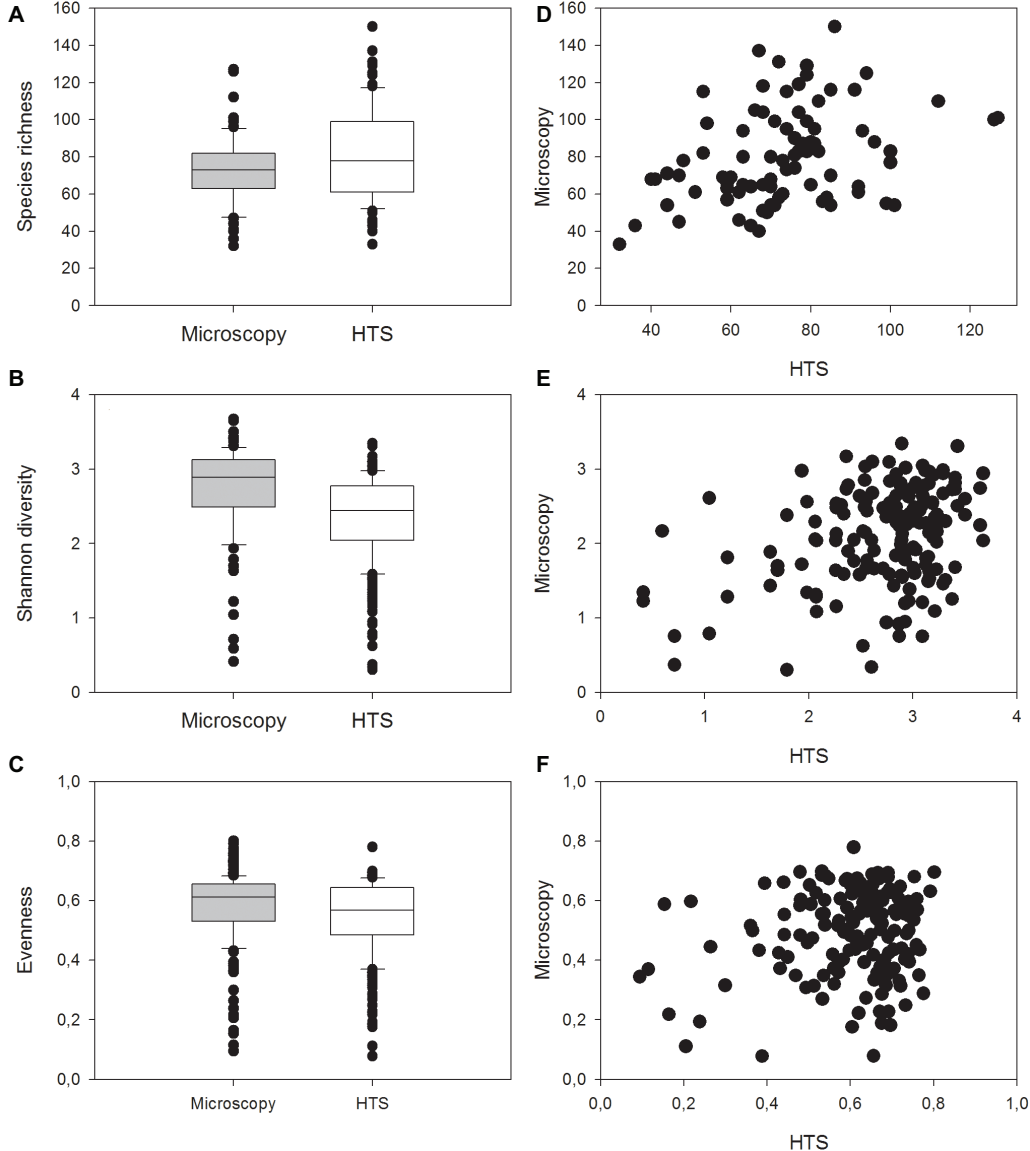
Median, standard deviation, 25 and 75% percentiles as well as outlier values of **(A)** richness (i.e., taxon richness), **(B)** Shannon diversity index, and **(C)** Evenness in the 83 lake water samples identified with light microscopy analysis and high-throughput sequencing (HTS) or with both the methods, and comparison of **(D)** species richness, **(E)** Shannon diversity index, and **(F)** Evenness between results obtained by high-throughput sequencing (HTS) and light microscopy (Microscopy).

In general, HTS revealed more genera belonging to the classes Cyanophyceae, Cryptophyceae and Dinophyceae, Bacillariophyceae, Mediophyceae, Euglenophyceae, and Synurophyceae (silica-scaled Chrysophyceae; Figure 5A). At the same time, light microscopy was able to identify more genera belonging to classes Chlorophyceae, Trebouxiophyceae, and Conjugatophyceae than HTS. In total, the number of genera identified by HTS was 340, while 196 genera were identified by light microscopy, and the number of shared genera, identified by both the methods, was 91. At species level, light microscopy analysis was able to identify more cyanobacteria, Chrysophyceaean, Bacillariophyceaean, Coscinodiscophyceaean, Chlorophyceaean, Trebouxiophyceaean, and Charophyceaean species (Figure 5B). In total, 266 species were identified by HTS, while 369 species were identified by light microscopy. Light microscopy analysis was able to identify more Chlorophyceaean, Conjugatophyceaean, Diatomophyceaean, and Xanthophyceaean species, while HTS identified more Synurophyceaean, Crytophyceaean, Dinophyceaean, and Raphidophyceaean species than light microscopy analysis. The number of shared species, identified by both methods, was only 40. Species richness (i.e., taxon richness) varied more in the HTS data than in microscopy data, but there was no significant difference in the richness between the two methods (Related-samples Wilcoxon signed rank test, *p* = 0.450; Figures 6A–F). The median Shannon diversity and the median Evenness were slightly lower in the HTS data, but the results were not consistent. Therefore, the differences in the indices between the methods were significant (Related-samples Wilcoxon signed rank test, *p* < 0.001 each).

**Figure 7:**
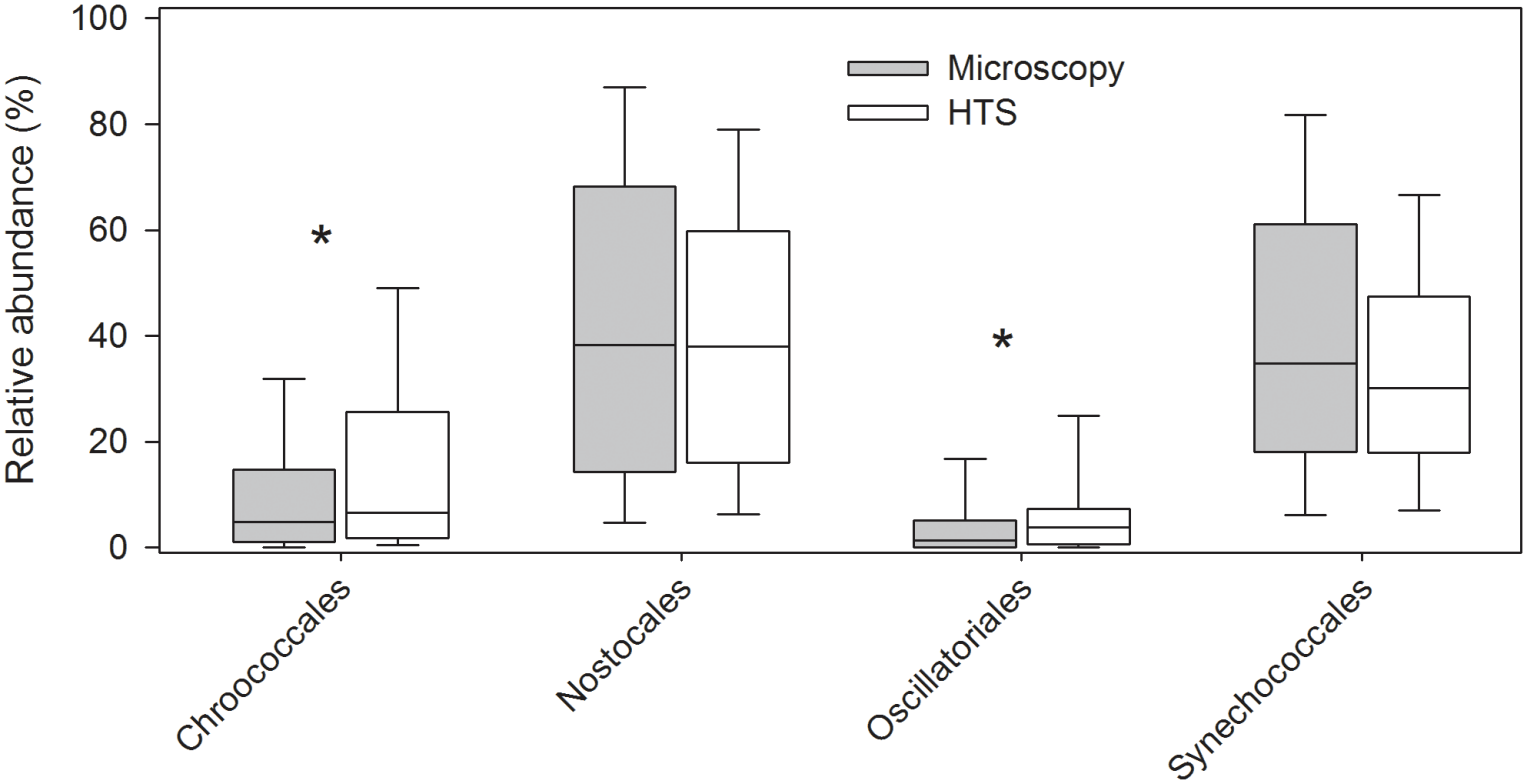
Relative abundances of cyanobacteria at order level analyzed by high-throughput sequencing (HTS) and light microscopy. The graph shows median, standard deviation, and 25 and 75% percentiles over the whole set of 83 lake samples. Significant differences between the light microscopy and HTS results are indicated with an asterisk (^*^) (Related-samples Wilcoxon signed rank test).

Pico-sized species (e.g., cyanobacterial genera *Synechococcus* and *Cyanobium*) and other small-sized species (e.g., Chlorophyceaean genera *Neochlorosarcina* and *Picocystis* and Chrysophyceaean genus *Chromophyton*), lacking morphological features required for identification by light microscopy, were detected by HTS. Also, identification of narrow (<2 μm in width) cyanobacterial filaments, or filaments with no visible cellular spacing or filaments which are otherwise very similar in morphology (e.g., *Planktothrix* and *Planktotricoides*) are difficult or impossible to identify at species (or even at genus) level by light microscopy. HTS was able to identify several such genera, as well as several benthic cyanobacterial genera, which are usually only temporarily present in plankton. Such benthic taxa are usually intentionally omitted from routine phytoplankton analysis (e.g., *Geitlerinema*, *Johanseninema*, *Desertifilum*, *Crinalium*, *Leptolyngbya*, *Limnolyngbya*, *Scytolyngbya* etc.). However, at order level, both the methods resulted in similar cyanobacterial community matrices (Mantel test, *r* = 0.41, *p* = 0.001; Figure 7, Supplementary Figure S2). Differences between the methods were significant for the cyanobacterial orders Nostocales and Synechococcales (related samples Wilcoxon signed rank test, *p* ≤ 0.001; Figure 7).

## Discussion

Traditionally, studies of the community composition of phytoplankton have been based on light microscopy analyses. However, phytoplankton analyses performed by light microscopy have some major problems. For example, identified taxa are rarely well documented (e.g., photographed) during analysis, Lugol-preserved phytoplankton samples last for only a few years, and durable preparations from most phytoplankton groups are often difficult or impossible and time-consuming to prepare. Furthermore, microscopy-based analyses are based on relatively small sample volumes (3–50 ml) and sub-counts are performed at several magnifications. When counts are done using higher magnifications, it is possible to count only a fraction of the total chamber area, and therefore cell counts are performed on a small portion of the sample volume.

An important advantage of genetic characterization of communities is that it generally allows for the analysis of larger sample volumes. This increases the likelihood of rare species, allowing more accurate detection of true diversity of phytoplankton. In addition, since sequencing results rely on robust genetic markers, sequences can be retained, and their affiliation can be refined later when more reference sequences are available in databases. Here, we applied the recently developed HTS method (Mäki and Tiirola, 2018) for SSU rRNA sequencing of phytoplankton samples. In this study, SSU rRNA was chosen as the target gene because it is an established molecular marker for phylogenetic identification of microbial communities (Woese, 1987; Vinje et al., 2014). Moreover, both the 16S and 18S rRNA sequences are available in databases, such as GenBank (Benson et al., 2013) and SILVA (Quast et al., 2012). The 16S and 18S rRNA genes are highly conserved, but variable regions allow phylogenetic reconstructions and identification of organism at different taxonomic levels (Pawlowski et al., 2012).

Our data from 83 lakes allowed comparisons of HTS and light microscopy methods to analyze the composition of phytoplankton community. Overall, cyanobacterial sequences were the most abundant in the HTS results. The proportion of cyanobacterial wet weight biomass, however, was considerably lower as compared to the phytoplankton total wet weight biomass obtained by light microscopy analysis. One reasonable explanation for the discrepancy between the proportions of cyanobacteria compared to eukaryotic phytoplankton in the HTS results is that eukaryotic phytoplankton are still poorly represented in the SILVA database compared to prokaryotic cyanobacteria. Therefore, there is an urgent need to improve 18S rRNA gene reference databases, in order to reach the conclusion of true community compositions by HTS. The excessive proportion of cyanobacteria in the HTS results compared to the microscopy results may also result from an incomplete ligation reaction of 18S rRNA and M13-RNA adapter, in which rRNAs must contain a 5′-end phosphate group. Although rRNAs are assumed to contain 5′-phosphate and lack the 5′-end capping (present in mRNAs of eukaryotes), a recent study showed that during nutritional depletion of yeast, newly synthesized rRNAs were 5′-end capped and resistant to 5′-phosphate-dependent exonuclease digestion (Fleischmann and Rocha, 2018). Although serial phosphorylation and dephosphorylation of rRNA precursors is a known phenomenon during maturation (Bruderer et al., 2003), the structure of mature 18S rRNAs can still include unknown properties and requires further studies to ensure equal ligation of eukaryotic and prokaryotic rRNA molecules. If the 5′-end of prokaryotic 16S rRNAs differs from eukaryotic 18S rRNAs, this could partly explain the small proportion of eukaryotic phytoplankton in the HTS results. Despite the disparity in the relative abundances of cyanobacteria and eukaryotic phytoplankton in the HTS results, the relative abundances of cyanobacteria at order level and eukaryotic phytoplankton at phylum and class level corresponded well between the methods.

The composition of identified taxa differed between the methods. In general, HTS revealed greater diversity at genus level. In contrast, more species were identified by conventional light microscopy, but the number of shared species, identified by both the methods, was low. Similar results were obtained by Xiao et al. (2014), when they sequenced the two hypervariable region of the rRNA (the V2 region of 16S gene and the V9 region of 18S rRNA gene). The HTS approach proved to be more robust in identification of cryptic species or species that are generally difficult to identify in water samples. Such are, for example, taxa whose species-level identification requires preparations (e.g., Diatomophyceae, Dinophyceae, and Synurophyceae). Furthermore, HTS was able to detect more genera and species that predominantly occur as single cells, e.g., many Cryptophyceaeans, Euglenophyceaeans, and Chlorophyceaeans, which may have fine morphological features that are not clearly visible with small inverted microscope objective magnifications (total magnification 1,000X or less). HTS was also able to identify taxa that tend to lose their cell shape when the samples are preserved with Lugol solution, e.g., many Raphidophyceans. HTS was also able to identify several narrow filamentous cyanobacterial genera that can only be identified at order level using light microscopy. The difference can also be due to the presence of taxa that are difficult or impossible to identify by light microscopy due to small size. The proportion of picocyanobacteria obtained by HTS corresponded with the epifluorescence microscopy results, although, the variation was high. The high variation was also observed by Ruber et al. (2018), who compared the results of flow cytometry and 16S rRNA gene sequencing in quantification of *Synechococcus* in German lakes.

The found differences in taxon composition and diversity between HTS and the microscopy results can be partly explained by the lack of corresponding sequences in the SILVA database. Many rare species or species that are currently not grown in cultures are missing from curated reference databases (e.g., Eiler et al., 2013; Xiao et al., 2014). Dynamic aquatic environments also generate morphological diversity (phenotypic diversity), causing intraspecies morphological variation, i.e. morphospecies with the same genotype (e.g., McManus and Katz, 2009). This may lead to overestimations of diversity, or to erroneous findings of cryptic species, e.g., within Dinophyceans (Thornhill et al., 2007). Lugol preservation of water samples may also affect the community composition results, since Lugol preservation is known to destroy some organelles important for the identification of Cryptophyceaean taxa by their morphological features (e.g., Xia et al., 2013) or even destroy some cells (e.g., Woelfl and Whitton, 2000).

Based on our results, HTS was better able to identify small (<2 μm) single-celled species, which cannot be distinguished into species or even into genus by light microscopy. However, HTS cannot yet replace light microscopy analyses, due to the lack of corresponding reference sequences in reference databases. We aligned our sequences against the SILVA database, which is considered to be the best curated available 16S and 18S database. Based on our results, especially Diatomophyceae Chlorophyceae, and Conjugatophyceae sequences are poorly represented in the database. Since only a fraction of all eukaryotic phytoplankton sequences are found in the reference databases and morphology-based identification of cryptic species is challenging, the community composition described by HTS is not the same as described by light microscopy (e.g., Luo et al., 2011). As more sequences are deposited to reference databases and problems with bioinformatics and taxonomic designation are being resolved, the method should be more comparable to the results of phytoplankton microscopy.

The consensus between microscopy and molecular results is not necessarily needed. HTS rRNA sequence data may already be sufficient in itself to monitor the environment. As such, the sequencing data can be used as a basis for environmental monitoring without linking sequences to taxonomy. However, the indicator values for DNA/RNA barcodes require a wide collection of environmental samples and metadata (see the opinion by Blaxter et al., 2005). In addition, the structure of the 5′-end of eukaryotic rRNAs should be resolved to confirm more robust application of the technique used in this study.

A major advantage of the chosen HTS primer-independent targeted metatranscriptomics method is the simultaneous identification and relative quantification of prokaryotic and eukaryotic community composition. The size range of such organisms ranges from several millimeters to less than 1 μm. Currently, different sampling methods, sample preservatives, and analytical methods are needed to study different groups of organisms. Ideally, all planktonic organisms could be assayed in a single water sample, without toxic or otherwise harmful preservatives and with a single assay. Furthermore, standardization of sample volume, for example, is easier with HTS, although filtering capacity may limit sample volumes in eutrophic waters. The primer-independent method can be further elaborated and refined for simultaneous, cost-effective environmental monitoring and status assessment, characterization of total diversity of all organisms and description of food web interactions or evaluation of restoration measures.

## Data Availability Statement

The sequence data analyzed in this study is publicly available in the National Center for Biotechnology Information, NCBI (SRA accession PRJNA577554). Requests to access the phytoplankton and picoplankton microscopy analysis datasets should be directed to KV (kristiina.vuorio@ymparisto.fi) or PS (pauliina.u.m.salmi@jyu.fi).

## Author Contributions

KV, MT, and AM took part in designing the study. PS and KV took part in the field work. KV performed phytoplankton microscopy, performed the comparison between HTS and microscopy results, and wrote the outline of the manuscript. KV and AM completed the manuscript. AM performed all molecular studies and did the bioinformatics. PS performed the picoplankton analysis. SA did the statistical analyses. All authors participated in writing and commenting and offered invaluable comments on the manuscript.

### Conflict of Interest

The authors declare that the research was conducted in the absence of any commercial or financial relationships that could be construed as a potential conflict of interest.

## Footnotes

^1^https://www.qiagenbioinformatics.com

## Supplementary Material

SUPPLEMENTARY FIGURE S1 Relative abundances of eukaryotic phytoplankton **(A)** sequences obtained by high throughput sequencing and **(B)** wet weight biomasses obtained by light microscopy at phylum level.

SUPPLEMENTARY FIGURE S2 Relative abundances of cyanobacterial **(A)** sequences obtained by high throughput sequencing and **(B)** wet weight biomasses obtained by light microscopy at order level.

## References

[ref1] Benson D. A. Cavanaugh M. Clark K. Karsch-Mizrachi I. Lipman D. J. Ostell J.. (2013). GenBank. Nucleic Acids Res. 41, D36–D42. 10.1093/nar/gks1195, PMID: 23193287 PMC3531190

[ref2] Blaxter M. Mann J. Chapman T. Thomas F. Whitton C. Floyd R. (2005). Defining operational taxonomic units using DNA barcode data. Philos. Trans. R. Soc. Lond. B Biol. Sci. 360, 1935–1443. 10.1098/rstb.2005.1725 16214751 PMC1609233

[ref3] Botero L. D’Imperio S. Burr M. McDermott T. R. Young M. Hasset D. J. (2005). Poly(A) polymerase modification and reverse transcriptase PCR amplification of environmental RNA. Appl. Environ. Microbiol. 71, 1267–1275. 10.1128/AEM.71.3.1267-1275.2005 15746328 PMC1065135

[ref4] Bruderer T. Tu L. Lee M. G. (2003). The 5′ end structure of transcripts derived from the rRNA gene and the RNA polymerase I transcribed protein coding genes in *Trypanosoma brucei*. Mol. Biochem. Parasitol. 129, 69–77. 10.1016/S0166-6851(03)00095-1, PMID: 12798508

[ref5] Carini P. Marsden P. J. Leff J. W. Morgan E. E. Strickland M. S. Fierer N. (2017). Relic DNA is abundant in soil and obscures estimates of soil microbial diversity. Nat. Microbiol. 2:16242. 10.1101/043372 27991881

[ref6] Carvalho L. Poikane S. Lyche Solheim A. Phillips G. Borics G. Catalan J. (2013). Strength and uncertainty of phytoplankton metrics for assessing eutrophication impacts in lakes. Hydrobiologia 704, 127–140. 10.1007/s10750-012-1344-1

[ref7] Cermeño P. Teixeira I. G. Branco M. Figueiras F. G. Marañón E. (2014). Sampling the limits of species richness in marine phytoplankton communities. J. Plankton Res. 36, 1135–1139. 10.1093/plankt/fbu033

[ref8] Cole J. J. (1982). Interaction between bacteria and algae in aquatic ecosystems. Annu. Rev. Ecol. Syst. 13, 291–314. 10.1146/annurev.es.13.110182.001451

[ref9] Cristescu M. E. (2014). From barcoding single individuals to metabarcoding biological communities: towards an integrative approach to the study of global biodiversity. Trends Ecol. Evol. 29, 566–571. 10.1016/j.tree.2014.08.001 25175416

[ref11] Eiler A. Drakare S. Bertilsson S. Pernthaler J. Peura S. Rofner C.. (2013). Unveiling distribution patterns of freshwater phytoplankton by a next generation sequencing based approach. PLoS One 8:e53516. 10.1371/journal.pone.0053516, PMID: 23349714 PMC3551911

[ref12] Eloe-Fadrosh E. Ivanova N. N. Woyke T. Kyrpides N. C. (2016). Metagenomics uncovers gaps in amplicon-based detection of microbial diversity. Nat. Microbiol. 4:15032. 10.1038/NMICROBIOL.2015.32 27572438

[ref13] EN 15204 (2006). Water quality. Guidance standard on the enumeration of phytoplankton using inverted microscope (Utermöhl technique).

[ref14] EN 16695 (2015). Water quality – Guidance on the estimation of phytoplankton biovolume.

[ref15] Fleischmann J. Rocha M. (2018). Nutrient depletion and TOR inhibition induce 18S and 25S ribosomal RNAs resistant to a 5′-phosphate-dependent exonuclease in Candida albicans and other yeasts. BMC Mol. Biol. 19, 1–8. 10.1186/s12867-018-0102-y 29351732 PMC5775620

[ref16] Guiry M. D. Guiry G. M. (2018). AlgaeBase. World-wide electronic publication. Galway: National University of Ireland. Available at: http://www.algaebase.org (Accessed December 27, 2018).

[ref17] Hajibabaei M. Shokralla S. Zhou X. Singer G. A. C. Baird D. J. (2011). Environmental barcoding: a next-generation sequencing approach for biomonitoring applications using river benthos. PLoS One 6:e17497. 10.1371/journal.pone.0017497, PMID: 21533287 PMC3076369

[ref18] Hering D. Borja A. Jones J. I. Pont D. Boets P. Bouchez A. (2018). Implementation options for DNA-based identification into ecological status assessment under the European water framework directive. Water Res. 138, 192–205. 10.1016/j.watres.2018.03.003 29602086

[ref19] Hillebrand H. Dürselen C.-D. Kirschtel D. Pollinger U. Zohary T. (1999). Biovolume calculation for pelagic and benthic microalgae. J. Phycol. 35, 403–424. 10.1046/j.1529-8817.1999.3520403.x

[ref20] Hillmann B. Al-Ghalith G. A. Shields-Cutler R. R. Zhu Q. Gohl D. M. Beckman K. B. (2018). Evaluating the information content of shallow shotgun metagenomics. mSystems 3:e00069-18. 10.1128/mSystems.00069-18 30443602 PMC6234283

[ref21] Houseley J. Tollervey D. (2009). The many pathways of RNA degradation. Cell 136, 763–776. 10.1016/j.cell.2009.01.019, PMID: 19239894

[ref23] Jost S. Medinger R. Boenigk J. (2010). Cultivation independent species identification of *Dinobryon* species (Chrysophyceae) by means of multiplex single-cell PCR. J. Phycol. 56, 901–906. 10.1111/j.1529-8817.2010.00871.x

[ref24] Karst S. M. Dueholm M. S. McIlroy S. J. Kirkegaard R. H. Nielsen P. H. Albertsen M. (2018). Retrieval of a million high-quality, full-length microbial 16S and 18S rRNA gene sequences without primer bias. Nat. Biotechnol. 36, 190–195. 10.1038/nbt.4045, PMID: 29291348

[ref25] Klindworth A. Pruesse E. Schweer T. Peplies J. Quast C. Horn M.. (2013). Evaluation of general 16S ribosomal RNA gene PCR primers for classical and next-generation sequencing-based diversity studies. Nucleic Acids Res. 41:e1. 10.1093/nar/gks808, PMID: 22933715 PMC3592464

[ref26] Komárek J. (2010). Cyanobacterial taxonomy: current problems and prospects for the integration of traditional and molecular approaches. Algae 21, 349–375. 10.4490/algae.2006.21.4.349

[ref27] Krienitz L. Bock C. (2012). Present state of the systematics of planktonic coccoid green algae of inland waters. Hydrobiologia 698, 295–326. 10.1007/s10750-012-1079-z

[ref28] Linnarsson S. (2010). Recent advances in DNA sequencing methods – general principles of sample preparation. Exp. Cell Res. 316, 1339–1343. 10.1016/j.yexcr.2010.02.036, PMID: 20211618

[ref29] Lodish H. Berk A. Zipursky S. L. Matsudaira P. Baltimore D. Darnell J. (2000). Molecular cell biology. 4th Edn. New York, NY, USA: W. H. Freeman.

[ref30] Longhurst A. Sathyendranath S. Platt T. Caverhill C. (1995). An estimate of global primary production in the ocean from satellite radiometer data. J. Plankton Res. 17, 1245–1271. 10.1093/plankt/17.6.1245

[ref31] Luo W. Bock C. Li H. R. Padisák J. Krienitz L. (2011). Molecular and microscopic diversity of planktonic eukaryotes in the oligotrophic Lake Stechlin (Germany). Hydrobiologia 661, 133–143. 10.1007/s10750-010-0510-6

[ref32] MacIsaac E. A. Stockner J. G. (1993). “Enumeration of phototrophic picoplankton by autofluorescence microscopy” in Handbook of methods in aquatic microbial ecology. eds. Kemp P. F. Sherr B. F. Sherr E. B. Cole J. J. (Boca Raton, FL: Lewis Publisher), 187–197.

[ref33] Mäki A. Salmi P. Mikkonen A. Kremp A. Tiirola M. (2017). Sample preservation, DNA or RNA extraction and data analysis for high-throughput phytoplankton community sequencing. Front. Microbiol. 8:1848. 10.3389/fmicb.2017.01848 29018424 PMC5622927

[ref34] Mäki A. Tiirola M. (2018). Directional high-throughput sequencing of RNAs without gene-specific primers. BioTechniques 65, 219–223. 10.2144/btn-2018-0082, PMID: 30284935

[ref35] McManus G. B. Katz L. A. (2009). Molecular and morphological methods for identifying plankton: what makes a successful marriage? J. Plankton Res. 10, 1119–1129. 10.1093/plankt/fbp061

[ref36] Neidhardt F. C. Magasanik B. (1960). Studies on the role of ribonucleic acid in the growth of bacteria. Biochim. Biophys. Acta 42, 99–116. 10.1016/0006-3002(60)90757-5, PMID: 13728193

[ref37] Padisák J. Borics G. Grigorsky I. Saróczki-Píntér E. (2006). Use of phytoplankton assemblages for monitoring ecological status of lakes within the water framework directive: the assemblage index. Hydrobiologia 553, 1–14. 10.1007/s10750-005-1393-9, PMID: 29018424

[ref38] Pawlowski J. Audic S. Adl S. Bass D. Belbahri L. Berney C.. (2012). CBOL protist working group: barcoding eukaryotic richness beyond the animal, plant, and fungal kingdoms. PLoS Biol. 10:e1001419. 10.1371/journal.pbio.1001419, PMID: 23139639 PMC3491025

[ref39] Porter T. M. Hajibabaei M. (2018). Scaling up: a guide to high-throughput genomic approaches for biodiversity analysis. Mol. Ecol. 27, 313–338. 10.1111/mec.14478 29292539

[ref40] Quast C. Pruesse E. Yilmaz P. Gerken J. Schweer T. Yarza P.. (2012). The SILVA ribosomal RNA gene database project: improved data processing and web-based tools. Nucleic Acids Res. 41, D590–D596. 10.1093/nar/gks1219, PMID: 23193283 PMC3531112

[ref41] Quince C. Walker A. W. Simpson J. T. Loman N. J. Segata N. (2017). Shotgun metagenomics, from sampling to analysis. Nat. Biotechnol. 35, 833–844. 10.1038/nbt.3935, PMID: 28898207

[ref220] R Core Team (2018). R: A language and environment for statistical computing. Vienna, Austria: R Foundation for Statistical Computing Available at: https://www.R-project.org/

[ref42] Reynolds C. (2006). The ecology of phytoplankton. Cambridge, UK: Cambridge University Press.

[ref43] Rodriguez-Ramos T. Dornelas M. Marañón E. Cermeño P. (2014). Conventional sampling methods severely underestimate phytoplankton species richness. J. Plankton Res. 36, 334–343. 10.1093/plankt/fbt115

[ref44] Ruber J. Geist J. Hartmann M. Millard A. Raeder U. Zubkov M. (2018). Spatio-temporal distribution pattern of the picocyanobacterium *Synechococcus* in lakes of different trophic state: a comparison of flow cytometry and sequencing approaches. Hydrobiologia 811, 77–92. 10.1007/s10750-017-3368-z

[ref45] Salmi P. Lehmijoki A. Salonen K. (2014). Development of picoplankton during natural and enhanced mixing under late-winter ice. J. Plankton Res. 36, 1501–1511. 10.1093/plankt/fbu074

[ref47] Shokralla S. Spall J. Gibson J. F. Hajibabaei M. (2012). Next-generation sequencing technologies for environmental DNA research. Mol. Ecol. 21, 1794–1805. 10.1111/j.1365-294X.2012.05538.x, PMID: 22486820

[ref48] Siegwald L. Touzet H. Lemoine Y. Hot D. Audebert C. Caboche S. (2017). Assessment of common and emerging bioinformatics pipelines for targeted metagenomics. PLoS One 12:e0169563. 10.1371/journal.pone.0169563, PMID: 28052134 PMC5215245

[ref49] Slomovic S. Laufer D. Geiger D. Schuster G. (2006). Polyadenylation of ribosomal RNA in human cells. Nucleic Acids Res. 34, 2966–2975. 10.1093/nar/gkl357 16738135 PMC1474067

[ref50] Sommer U. Padisák J. Reynolds C. S. Juház-Nagy P. (1993). Hutchinson’s heritage: the diversity-disturbance relationship in phytoplankton. Hydrobiologia 249, 1–7. 10.1007/BF00008837

[ref51] Thornhill D. J. Lajeunesse T. C. Santos S. R. (2007). Measuring rDNA diversity in eukaryotic microbial systems: how intragenomic variation, pseudogenes, and PCR artifacts confound biodiversity estimates. Mol. Ecol. 16, 5326–5340. 10.1111/j.1365-294X.2007.03576.x, PMID: 17995924

[ref52] Utermöhl H. (1958). Zur Vervollkommnung der quatitativen Phytoplanktonmethodik. Int. Ver. Theor. Angew. Limnol. Mitt. 9, 1–38.

[ref53] Valentini A. Taberlet P. Miaud C. Civade R. Herder J. Thomsen P. F.. (2016). Next-generation monitoring of aquatic biodiversity using environmental DNA metabarcoding. Mol. Ecol. 25, 929–942. 10.1111/mec.13428, PMID: 26479867

[ref54] Vinje H. Almoy T. Liland K. H. Snipen L. (2014). A systematic search for discriminating sites in the 16S ribosomal RNA gene. Microb. Inform. Exp. 4:2. 10.1186/2042-5783-4-2 24467869 PMC3910680

[ref55] Willén E. (1976). A simplified method of phytoplankton counting. British J. Phycol. 11, 265–278.

[ref56] Woelfl S. Whitton B. A. (2000). Sampling, preservation and quantification of biological samples from highly acidic environments (pH ≤3). Hydrobiologia 433, 173–180. 10.1023/A:1004099527441

[ref57] Woese C. R. (1987). Bacterial evolution. Microbiol. Rev. 51, 221–271. PMID: 2439888 10.1128/mr.51.2.221-271.1987 PMC373105

[ref58] Xia S. Cheng Y. Zhu H. Liu G. Hu Z. (2013). Improved methodology for identification of Cryptomonads: combining light microscopy and PCR amplification. J. Microbiol. Biotechnol. 23, 289–296. 10.4014/jmb.1203.03057, PMID: 23462000

[ref59] Xiao X. Sogge H. Lagesen K. Tooming-Klunderud A. Jakobsen K. S. Rohrlack T. (2014). Use of high throughput sequencing and light microscopy show contrasting results in a study of phytoplankton occurrence in a freshwater environment. PLoS One 9:e106510. 10.1371/journal.pone.0106510, PMID: 25171164 PMC4149573

