# Supplementary figures and images for: Consistency of Targeted Metatranscriptomics and Morphological Characterization of Phytoplankton Communities
Source: Front Microbiol. 2020 Feb 6;11:96. doi: 10.3389/fmicb.2020.00096 (PMC7016081; doi:10.3389/fmicb.2020.00096)

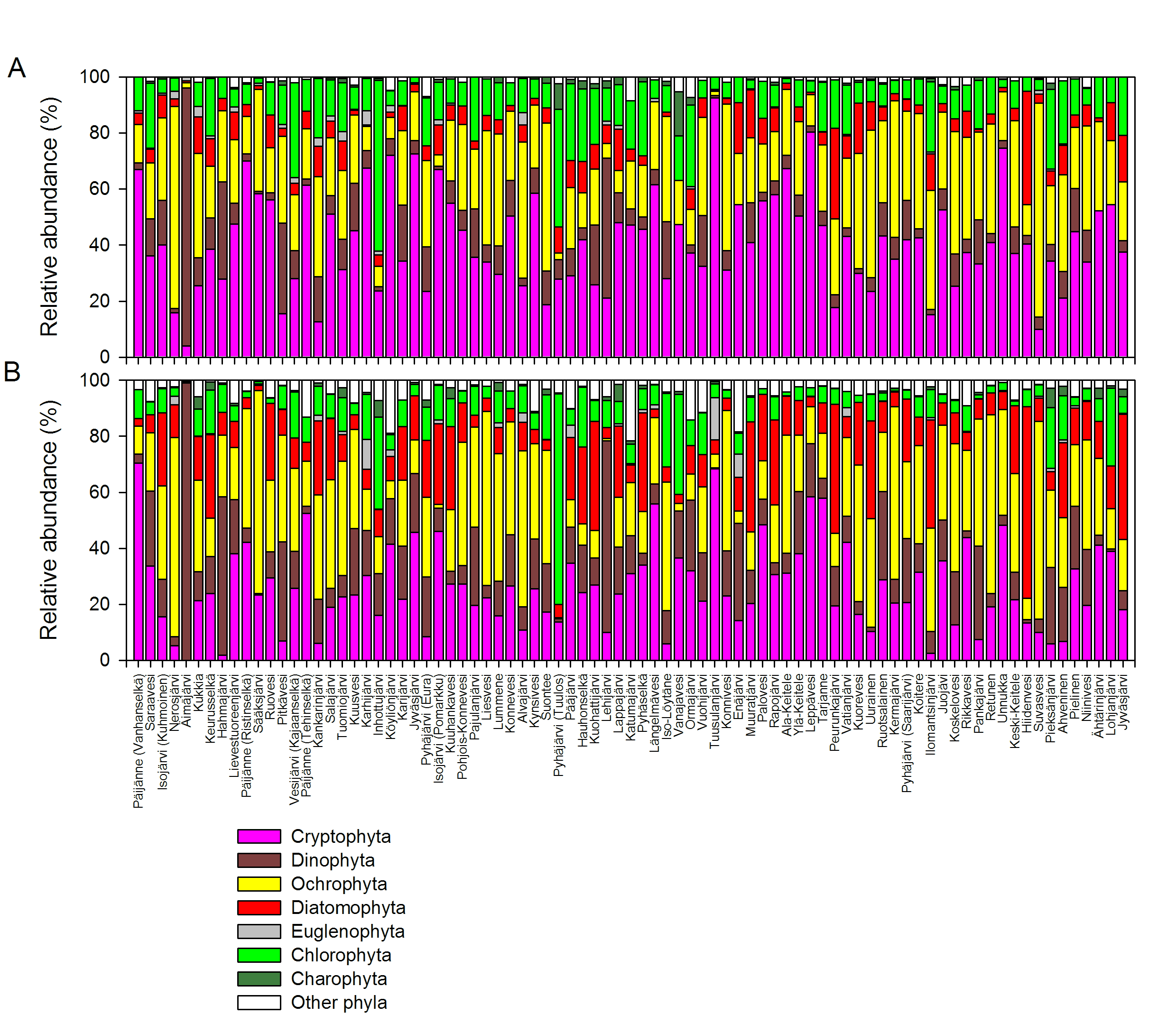

Supplement: SUPPLEMENTARY FIGURE S1 — Relative abundances of eukaryotic phytoplankton (A) sequences obtained by high throughput sequencing and (B) wet weight biomasses obtained by light microscopy at phylum level. [file Image_1.TIF]

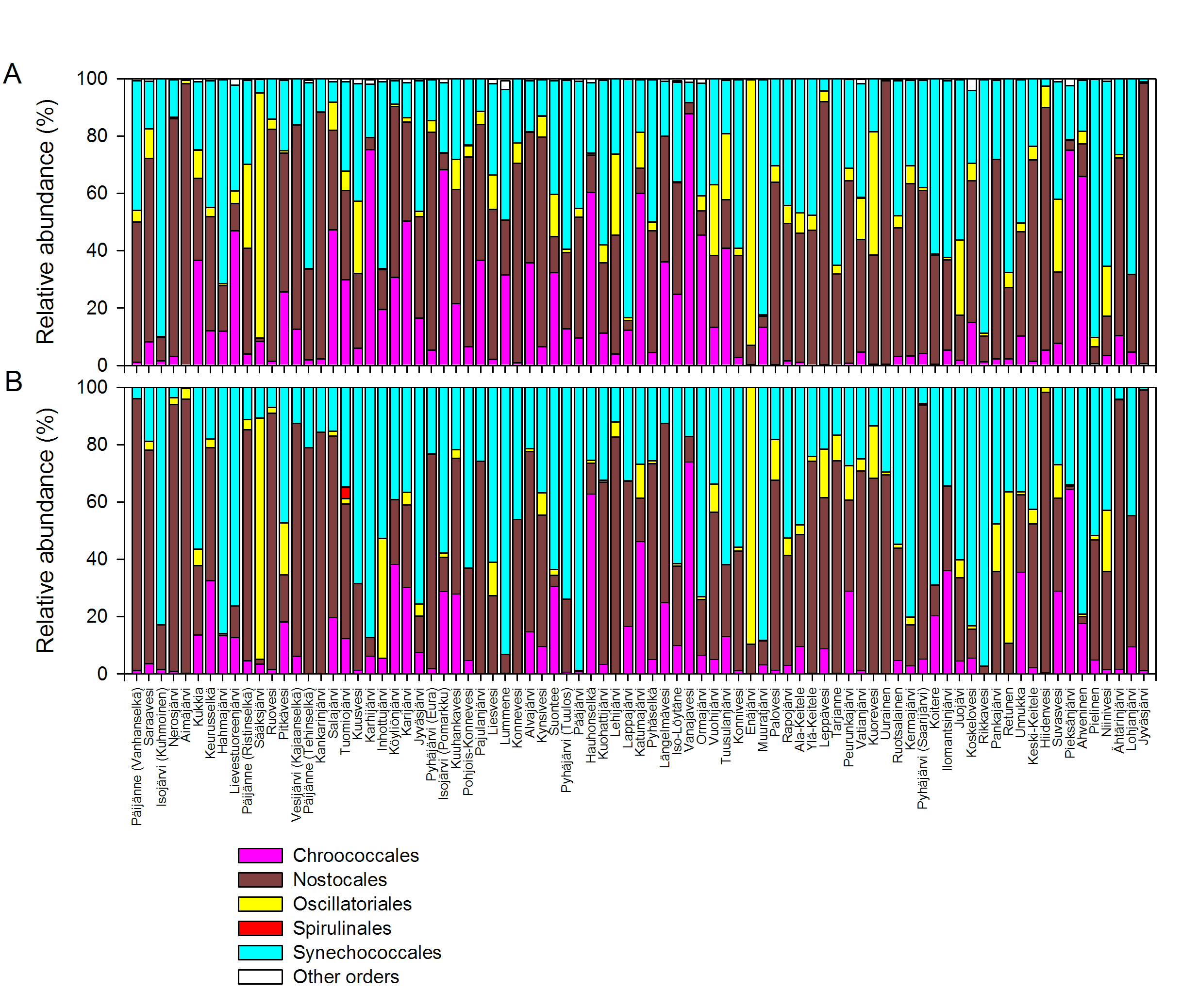

Supplement: SUPPLEMENTARY FIGURE S2 — Relative abundances of cyanobacterial (A) sequences obtained by high throughput sequencing and (B) wet weight biomasses obtained by light microscopy at order level. [file Image_2.TIF]
